# Sustainable management of the potato cyst nematode, *Globodera rostochiensis*, with two microbial fermentation products

**DOI:** 10.3389/fpls.2022.987059

**Published:** 2022-10-05

**Authors:** Anusha Pulavarty, Ankit Singh, David Smyth, Jai Prakash Mehta, Karina Horgan, Thomais Kakouli-Duarte

**Affiliations:** ^1^Molecular Ecology and Nematode Research Group, enviroCORE, Department of Applied Science, South East Technological University (SETU), Carlow, Ireland; ^2^Alltech Bioscience Centre, Dunboyne, County Meath, Ireland

**Keywords:** potato cyst nematodes, *Globodera rostochiensis*, MFP5075, MFP3048, Alltech

## Abstract

Potato cyst nematodes (PCN) cause an overall 9% yield loss of total potato production worldwide. Research on sustainable management of PCN is still under progress. Two microbial fermentation products (MFPs) from Alltech, a proprietary blend formulated with a bacterial fermentation media and a Cu component (MFP5075), and a microbial based product (MFP3048), were evaluated against the PCN *Globodera rostochiensis*. In laboratory tests, effectiveness of the MFPs was recorded in terms of PCN juveniles (J2) hatching from cysts, J2 mortality and their attraction toward potato roots using pluronic gel. Greenhouse trials were conducted to study the effect of the products on PCN infestation in potato plants and a pilot scale experiment was conducted to study the impact of these MFPs on nematode biodiversity in garden soil. All treatments were performed within a concentration range of 0, 0.5, 1, and 2% (v/v) MFP5075 and 2, 6, 10, and 20 g/10 ml (w/v) MFP3048. The attraction assay, juvenile hatching and the PCN infestation in plants results were compared with those in an untreated control and a commercial nematicide (Nemguard™) treatment. After 24 h of treatment with 0.5 and 1% MFP5075, a 13-fold and 43-fold reduction, respectively, relative to J2 survival was recorded compared to that of untreated control. However, no J2 survived at 2% and above concentration of the MFP5075 treatment. Treatment with MFP3048 was effective in causing mortality of J2 only after 48-h. In the attraction assay, a 20-fold and 8-fold reduction in number of J2 attracted toward potato roots was observed, when treated with MFP5075, compared to the untreated and the Nemguard™ treatment, respectively. Subsequently, 30–35 PCN cysts were treated with both products dissolved in potato root diffusate and the results were recorded in terms of number of J2 hatched in each treatment after 10 days. No J2 hatched in the MFP5075 treatment, whereas mean numbers (±SE) of 243 ± 11.5, 30 ± 2.5, and 1.3 ± 0.6 J2 were noted in the untreated control, MFP3048, and the Nemguard™ treatment, respectively. The treatment with the MFPs compromised the integrity of the unhatched J2, which looked granular, whereas the internal organs of the unhatched J2 could be clearly identified in the untreated control. In plant infestation studies, treatment with MFP3048 and MFP5075 caused 90.6 and 84.9 percent reduction in PCN infestation, respectively, in terms of cysts developed on roots compared to untreated control. Overall, results indicate that the MFPs could potentially provide a promising alternative for sustainable PCN management.

## Introduction

Potatoes are one of the most important non-grain food crops in the world with an annual production of approximately 314,140,107 tons/year. Potatoes can be grown in varied climatic conditions and take less time and input to grow. They are considered to be an essential food security crop by United Nations due to their nutritional value, high food energy and complex carbohydrate content per unit of land (Wijesinha-Bettoni and Mouillé, 2019). China is the top potato producer in the world with 90,321,442 tonnes produced per year, followed by India (48,529,000 tonnes yearly), Ukraine, Russian Federation, United States, Bangladesh, Germany and France.^1^ With an increasing world population and urbanization, there is a global increase in potato consumption and therefore there is a high demand for its production (Price et al., 2021). Furthermore, there is a huge demand from the fast-food industry for processed potatoes (e.g., crisps, starch, and chips) due to huge economic returns (Abong and Kabira, 2013). Various recent reports highlight the importance of global potato production and limitations faced by growers due to increasing demands (Mburu et al., 2020; Gartner et al., 2021; Price et al., 2021; Pulavarty et al., 2021b).

Over the last 50 years, in terms of yield development, potatoes (46.2%) lag behind other crops such as corn (158.8% increase), wheat (135.5%), rice (109.6%), and soy (94.6%) (Redcliffe and Australia, 2021). This could be attributed to various pests infesting the potato crops globally, improper cropping practices and substandard tuber quality (Kaguongo et al., 2014). Some of the important diseases, disorders or defects associated with potato crop are early blight, late blight, root knot nematodes, potato cysts nematodes, fusarium rot, ring rot, pink rot, fusarium wilt, verticillium wilt, blackspot, blackheart, and leafroll (Olivier et al., 1998). *Globodera* spp*., Meloidogyne* spp*., Pratylenchus spp. and Trichodorus* spp. are reported to be some of the major nematode pests infecting potato plants (Nicol et al., 2011).

Potato cyst nematodes (PCN; *Globodera rostochiensis* and *Globodera pallida*) are such economically important pathogens causing huge crop losses in potato crop worldwide (De Boer et al., 1996; Aires et al., 2009; Niere and Unger, 2012; Eche et al., 2018; Mburu et al., 2020; Price et al., 2021; Ochola et al., 2022). Jones et al. (2013) have reported PCN as one of the top ten plant parasitic nematodes (PPN) based on their economic and scientific importance. They are known to cause enormous crop losses and various researchers have reported decline in potato yields in various parts of the world, such as in Kenya (Mwangi et al., 2015; Mburu et al., 2020), Rwanda (Niragire et al., 2019), Uganda (Cortada et al., 2020), North America (Dandurand et al., 2019), United Kingdom (Gartner et al., 2021), India (Chandel et al., 2020), South Korea (Kwon et al., 2018), China (Zhang et al., 2021), Iran (Hajihassani et al., 2013), Columbia (Vallejo et al., 2021), and Switzerland (Ruthes and Dahlin, 2022). Due to their wide spread and prolonged survival in soil (upto 20 years) and resistance to extreme temperatures it is tough to permanently eradicate PCN (Chandel et al., 2020).

Applications of synthetic chemical nematicides are expensive and have known to cause various environmental issues and a long term impact on human health (Douda et al., 2021). Many chemical nematicides have been therefore banned in the recent past (Pulavarty et al., 2021b). The biofumigation approach has been practiced in the past few years to manage PCN, however, the efficacy of the fumigant deteriorates over time due to various environmental factors such as temperature, humidity and soil characteristics (Hajihassani et al., 2018).

Some research groups have reported the application of extracts from Brassicaceae plants (Aires et al., 2009; Lord et al., 2011; Ngala et al., 2015), but, the application of such extracts have been reported to cause root decay and eventually lead to rotting of the plant biomass resulting in unpleasant odors in the fields (Ngala et al., 2015). In most cases, with the application of various products there is a lack of information on the effect of those products on other beneficial soil microbes and soil biodiversity. A thorough investigation on application of any product on beneficial nematodes, fungi, bacteria, or soil biodiversity as a whole is essential for a sustainable PCN management.

Recently, a most successful wrap-and-plant technology was reported by a research group in East Africa, which uses banana-matrix as a seed wrap to control the *G. rostochiensis* infestation (Ochola et al., 2022). This approach has proven very successful to sustainably manage PCN and other crop pests while increasing the potato yields significantly. However, large scale application of this technology needs to be validated and confirmed on larger scale production. Despite much international collaborative research and significant efforts, PCNs still continue to manifest vast damage to potato crops universally.

The current study aims to assess the potential of two microbial fermentation products, a proprietary blend formulated with a bacterial fermentation media and a Cu component (MFP5075), and a microbial based product (MFP3048), against the PCN *G. rostochiensis*. *In vitro* laboratory experiments were conducted to study the effect of MFPs on juvenile (J2) mortality, J2 hatching from the cysts, granulation of unhatched eggs and attraction of J2 toward potato roots. Subsequently, plant trials were conducted in greenhouse conditions to study the efficacy of the MFPs in controlling *G. rostochiensis* infestation in potato plants. The effect of MFPs on PCN, in terms of laboratory bioassays and greenhouse trials were compared with those of the untreated control and a commercial organic nematicide (Nemguard™) treatment.

The nematode biodiversity in soil after application of the MFPs was compared with that of biodiversity in the untreated garden soil and treatment with two commercial nematicides, Nemguard™ (organic), and Vydate™ (synthetic).

## Materials and methods

### Sourcing of potato cyst nematodes cysts and hatching of *Globodera rostochiensis* juveniles (J2)

*Globodera rostochiensis* cysts were received as a kind offer from Dr. Colin C. Fleming, Grassland and Plant Science Branch, Agri-Food and Biosciences Institute, Belfast, United Kingdom. The cysts were incubated at 4°C to break their dormancy for 6 months. After 6 months, a hatching assay was performed by incubating the cysts in potato root diffusate (PRD) to check their viability. Subsequent experiments were all performed only after ensuring the viability of the cysts.

### Collection of potato root diffusate

Potato rood diffusate was collected following protocol described by Turner et al. (2009) with the following modifications: tubers of susceptible variety, *Désirée*, were grown in a one liter soil pot, and the plants were allowed to grow until they reached an approximate height of 15 cm and roots were seen growing out from the base of the pots. These plants were then used for PRD collection. On the day of collection, the plants were not watered. One hundred milliliters of distilled water was gradually added per pot until the soil was saturated and a pale brown flow through liquid leached out from the bottom of the pot. A plastic saucer was placed underneath to collect the initial flow through liquid. The collected liquid was added back into the pot and was let drain for a second time. This process was repeated two more times. The liquid flow through that was collected in the saucer was poured into an amber colored glass bottle. A 1 month old potato plant was then kept at 25°C in the same glass bottle with the roots submerged in the flow through liquid for 24 h. Subsequently, the PRD was obtained by filtering the flow through liquid through a 90 μm sieve to remove the soil particles. The diffusates were then stored at 4°C until use.

### Bioassay: Experimental design and microbial fermentation products treatment to determine lethal concentrations and doses

Both products were tested initially on beneficial entomopathogenic nematodes (EPN; Pulavarty et al., 2020) and results indicated that 7% MFP5075 was considered as LC50 concentration and no mortality was noted when treated with MFP3048 (Pulavarty et al., 2020). These findings also revealed that at 4% MFP5075 and below there was no mortality caused to EPN (Pulavarty et al., 2020), therefore, all subsequent treatments were performed below 4% to ensure no harm is caused to EPN in the soil. The products were also reported to reduce *Meloidogyne javanica* infestation in tomato plants when treated with 1–3% MFP5075 and also when combined with 3 g MFP3048 (Pulavarty et al., 2021a).

*Globodera rostochiensis* cysts were hydrated in distilled water for 24 h. After 24 h, the cysts were incubated in PRD for 1 week. First batch of J2 obtained after 1 week of incubation were discarded and the cysts were placed into new petri dishes containing fresh PRD for another few days to collect J2 that were used for the bioassays.

All the treatment studies were conducted in 96-well plates with 10 replicates per treatment concentration. Freshly cultured J2, no older than 2 days post-cyst emergence, were used for the study. In each well, 200 μL of PRD containing approximately 20–30 J2 and various product (MFP5075 and MFP3048 Alltech ACS, Dunboyne, Co. Meath, Ireland) dilutions were added to obtain the required concentration. Potato root diffusate (200 μl) containing J2 without any product was considered as control. The concentration range for MFP5075 was 0–2% and for MFP3048 was 0–20 g dissolved in 10 ml of water. MFP5075 was directly pipetted in to 200 μl PRD in 96-well plate to obtain 0.5, 1, and 2% v/v, respectively. MFP3048, a powdered product, was prepared by adding 2, 6, 10, and 20 g in 10 ml w/v of distilled water in a falcon tube. These tubes containing MFP3048 product were kept in the incubator shaker for 24 h before use. After 24 h, 100 μl supernatant from each of the falcon tubes containing 2, 6, 10, and 20 g in 10 ml was added into each well to obtain an concentration range of 1, 3, 5, and 10 g per 10 ml, respectively.

After 24 h incubation at 20–22°C, survival and mortality were calculated by counting the motile and immotile J2 using an Olympus Stereo Microscope (SZX7). The mortality of immotile J2 was ensured by gently touching them with a needle. Another set of plates with the same concentration range was analyzed after 48 h incubation. It was observed that in the MFP5075 treatment, the mortality rate was similar to that of 24 h incubation. Therefore, data recorded after 24 h incubation were considered for statistical analysis for the MFP5075 treatment. However, in the MFP3048 treatment, J2 mortality occurred only after 48 h treatment, therefore, data recorded after 48 h incubation was considered for statistical analysis. All these bioassays were repeated thrice to ensure the reproducibility of the results.

### Attraction assays using 20% (w/v) pluronic gel

Fifty milliliters of a 20% (w/v) pluronic gel PF-127 (Sigma, St. Louis, MO, United States) solution was prepared in distilled water (Sasaki-Crawley et al., 2012) and the dissolved gel was stored at 4°C until use. Attraction assays were performed on glass slides using a protocol reported by Pulavarty et al. (2021a). Approximately 40–50 J2 were applied onto each slide containing pluronic gel solution (300 μl) in 10 mM sodium phosphate and PRD (200 μl). A 1-month-old potato root tip, 1 cm in length, was placed on the center of the glass slide containing the gel-nematode mixture. Ten microliters of MFP5075 (1%) was added to three of the glass slides, ten microliters of MFP3048 (20 g/10 ml) was also added to another set of three slides. Three slides containing the gel-nematode mixture without any products was regarded as the negative control (untreated). Three more slides with the gel-nematode mixture treated with 10 microliters of Nemguard™ (20 Kg/hectare; which corresponds to 13.3 mg/L) was considered a positive control for the experiment.

The potato root tip on each slide was covered with a transparent cover slip to prevent evaporation and drying of liquid and to clearly visualize movement of J2 toward potato roots. Individual slides were all incubated at room temperature, and the movement of J2 in each of the slides was closely monitored after every 10 min, upto 60 min, using an Olympus Stereo Microscope (SZX7). After 60 min of treatment, the mean number of J2 attracted toward the potato root in each slide was noted and the data recorded was used for statistical analysis. All the treatments including positive and negative controls were repeated nine times over a span of 1 week with three replicates each time (total of 27 replicates/treatment) to ensure the reproducibility of the results. The mean values obtained were used for statistical analysis.

### *Globodera rostochiensis* juvenile hatching assay from the cysts

*Globodera rostochiensis* cysts after incubation at 4°C for a period of 6 months were used for this assay. Approximately, 30–35 cysts were placed in individual petridishes (30 mm × 15 mm) for treatment with MFP5075 (2%), MFP3048 (20 g/10 ml), and Nemguard™ (13.3 mg/L) dissolved in PRD. The Petri dish containing only PCN cysts and PRD without any product were considered negative controls. Mean number of PCN J2 hatched in each petri dish was manually recorded after every 7 days upto a period of 4 weeks. After each week, all the J2 were removed in each petri dish and were replaced with fresh PRD containing product’s dilutions to continue the assay. The mean number of J2 recorded after 4 weeks of treatment was used for statistical analysis.

After 4 weeks of treatment with various products, some cysts were carefully broken open to study the granulation of unhatched eggs in the treated and untreated conditions (Feist et al., 2020). Individual cysts were removed from the treatment solutions, washed with ddH_2_O and transferred on to a glass slide containing a few drops of ddH_2_O. Cysts were carefully broken open with scalpel and tweezers without causing any damage to the eggs. Juvenile integrity within each unhatched egg was observed under the microscope. The total number of unhatched and granular eggs were enumerated in the treated and control groups. The unhatched eggs were considered as “granular,” where the mouth part and tail of the enclosed J2 was not distinguishable and appeared dark. The percentage of granular unhatched eggs in each cyst was calculated as: (Number of granular unhatched eggs)/(Total number of unhatched eggs) × 100. Data was represented as the percentage of unhatched eggs that appear granular per cyst in each treatment and control group.

Some of the cysts, after 4-weeks of treatment with MFP3048, MFP5075, and Nemguard™, were transferred back into individual wells of the 24 -well plates, containing normal PRD without any products to record the J2 hatching in absence of the products. After 10 days, number of J2 in each well were manually counted under the stereoscope.

### Effect of microbial based products on *Globodera rostochiensis* infestation in potato plants

New potato *Solanum tuberosum* Désirée tubers were collected from Teagasc, Oak Park, Carlow and exposed to cold spells at 4°C with high humidity for 2 months. Subsequently, the tubers were shifted to a dark room with 20–22°C and high humidity to encourage their germination. Within a period of 2 weeks sprouting was observed on the tubers. Immediately the small sprouted eye regions were gently excised from the tuber using a melon scoop and were potted on to small plastic pots containing thoroughly dried and sterilized sand: soil mixture 90:10. These potato tubers were maintained in an environmentally controlled glasshouse conditions at 32 ± 2°C, 70 ± 10% relative humidity (RH), and natural 14 h day/10 h night cycle, until the seedlings attained a height of 10–15 cm. Then, the nematode bioassays were performed to study the effect of the MFPs on PCN infestation in potato plants:

Prophylatic treatment: For this study, the individual 1 month old potato seedlings were first treated with MFP5075 (2%), MFP3048 (20 g/10 ml), and Nemguard™ (13.3 mg/L) dissolved in water. After treatment for a period of 2 weeks, each seedling was infected with approximately 500–600 freshly hatched *G. rostochiensis* J2. Plants with only infection and no treatment were considered as inoculated untreated control. After infection, the potato seedlings were maintained in an environmentally controlled plant growth room at 32 ± 2°C, 70 ± 10% relative humidity (RH), and natural 14 h day/10 h night cycle. Treatments, including control pots, were set up in triplicate and were allowed to grow for a period of 60 days. After the treatment duration of 60 days, the potato plants were harvested to record the various growth parameters in terms of shoot height (SH; cm), root length (RL; cm), fresh weight (FW; g), dry weight (DW; g), and number of leaves (NL). SH and RL were measured using a meter ruler, FW and DW were recorded using laboratory weighing scales, NL was enumerated by visual counting. The number of the new cysts or females produced in each root system was used as a measure to study the impact of the MFPs on PCN infestation. For this, the potato roots were carefully observed to manually count the number of females developed on each root system. The results were compared with those of the inoculated untreated control plants and with those of commercial nematicide (Nemguard™) treatment. All experiments were repeated three more times over a period of 6 months to ensure the reproducibility of the results.

### Garden soil treatment with microbial fermentation products, deoxyribonucleic acid extraction, and sequencing

**FIGURE 1:**
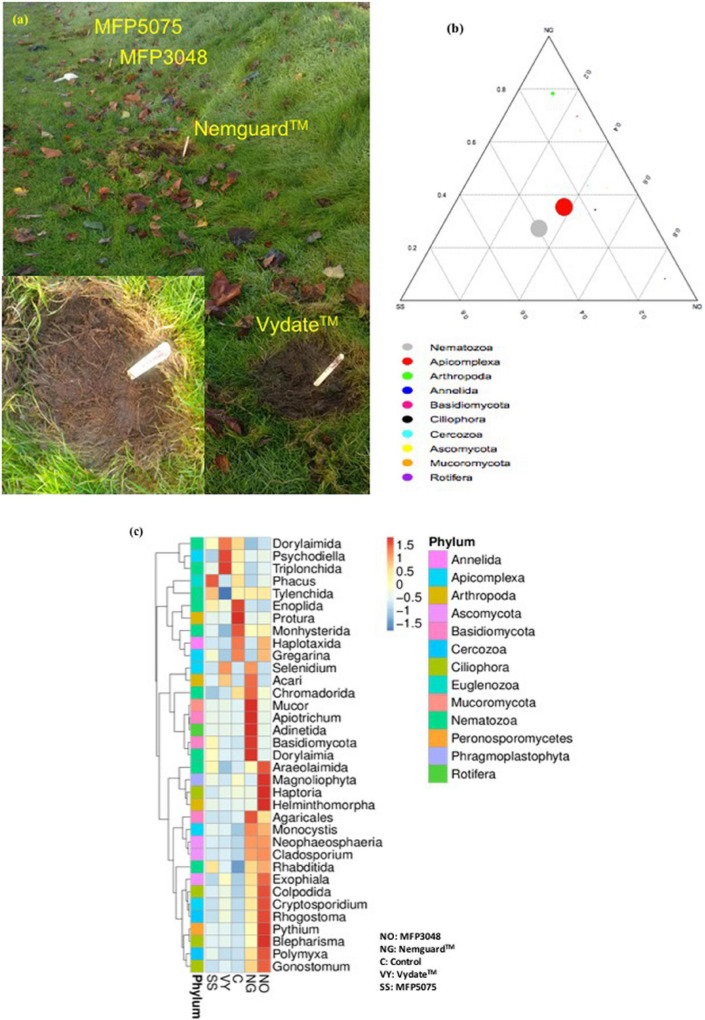
**(a)** Garden patches showing treatments with different products; **(b)** Ternary plot; **(c)** Heatmaps representing the abundances of the top nine nematode orders in treatment groups. The Z-score ranges between 1.5 and –1.5 and represents the distance between the raw score and the mean of the standard deviation. “Z” is negative when the raw score is below the mean, and vice versa.

A pilot scale experiment was conducted to study the impact of the MFPs on nematode biodiversity in untreated garden soil. For this study a random plot within the Kilkenny Road campus of SETU was chosen. In that, an individual garden patch as shown in Figure 1a of approximately, 45 cm × 45 cm size, was treated with the following: MFP5075 (2%), MFP3048 (20 g/10 ml), Nemguard™ (20 Kg/hectare), and Vydate™ (55 Kg/hectare) dissolved in water. A patch without any treatment was considered as untreated negative control. Treatment with Nemguard™, a commercial organic nematicide and Vydate™, a commercial synthetic chemical nematicide were considered as positive controls (Figure 1a). The individual products were mixed uniformly in the soil upto a depth of 20 cm. After 15 days, soil samples (approximately 25 g) were freshly collected from each patch. A total of 30 samples were obtained (5 treatments X 6 replicate samples per plot) and sieved through a 2 mm mesh. An individual soil sub-sample was added to individual centrifuge tubes containing 25-ml of deionized water and centrifuged for 10 min at 3500 rpm. The supernatant was discarded and the residue was dried overnight at room temperature (Karpinska et al., 2021). Subsequently, from the 0.25 g of soil sub-samples per treated patch, total soil deoxyribonucleic acid (DNA) was extracted using the Qiagen DNeasy^®^ PowerSoil^®^ Pro kit, as per the manufacturer instructions. Before outsourcing to Novogen Co., Ltd., the total DNA was quantified using both Invitrogen™ Qubit 4 Fluorometer and NanoDrop™ and its quality and integrity was measured by performing agarose gel electrophoresis.

The nematode18S V4 rRNA region was sequenced using the MN18F (5′CGCGAATRGCTCATTACAACAGC 3′) and 22R (5′GCCTGCTGCCTTCCTTGGA 3′) primer pairs, on an Illumina paired-end platform (Bhadury et al., 2006; Karpinska et al., 2021). The data obtained from the sequencing company was studied and analyzed to understand the effect of the MFPs on nematode biodiversity in comparison to the untreated control soil and that treated with commercial nematicides.

Paired-end reads were assigned to samples based on their unique barcodes and truncated by cutting off the barcode and primer sequences. Paired-end reads were merged using FLASH (Magoè and Salzberg, 2011), which was designed to merge paired-end reads when at least some of the reads overlap with the read generated from the opposite end of the same DNA fragment; the splicing sequences were called raw tags. Quality filtering on the raw tags was performed under specific filtering conditions to obtain the high-quality clean tags (Bokulich et al., 2013) according to the Qiime (V1.7.0) quality control process. The tags were compared with the reference database (SILVA138 database^2^) using UCHIME algorithm (UCHIME Algorithm^3^) (Edgar et al., 2011) to detect chimera sequences^4^ and the chimera sequences were removed (Haas et al., 2011) leaving the effective tags.

Sequences analysis were performed by Uparse software (Uparsev7.0.1090^5^) (Edgar, 2013) using all the effective tags. Sequences with ≥97% similarity were assigned to the same OTUs and representative sequences for each OTU were screened for further annotation. For each representative sequence Qiime (Version 1.7.0^6^) (Altschul et al., 1990) in Mothur method was performed against the SSUrRNA database of SILVA138 database^7^ (Wang et al., 2007) for species annotation at each taxonomic rank (Threshold: 0.8∼1) (Quast et al., 2013) (kingdom, phylum, class, order, family, genus, species).

To obtain the phylogenetic relationship of all OTUs, the MUSCLE (Edgar, 2004) (Version 3.8.31^8^) compared multiple sequences rapidly.

Operational taxonomic units abundance information were normalized using a standard of sequence number corresponding to the sample with the least sequences. Subsequent analysis of alpha diversity and beta diversity were all performed based on this output normalized data.

### Statistical analysis

All the experiments were statistically designed and analyzed. The experiments were arranged in a completely randomized factorial design (CRD). The results recorded from the MFP treatments in terms of PCN J2 survival, hatching assays, pluronic gel attraction assays, granulation of unhatched eggs and the plant-PCN trials were subjected to analysis of variance (ANOVA), using IBM-SPSS, version 23. Three sets of experimental plant trials in prophylactic treatments were performed over a span of 6 months to confirm reproducibility of results. Each trial had three replications per concentration including the untreated controls. Analysis of Variance was conducted for each experimental trial separately and the results from each trial were subjected to factorial ANOVA, to determine if the data was similar. Due to no significant difference among the trials, results presented in this study were the mean values of all the trials combined. Treatment means were separated using least significant differences (LSDs) obtained from analysis of variance (ANOVA) using IBM SPSS version 23.

## Results

### Bioassay: Experimental design and microbial fermentation products treatment to determine lethal concentrations and doses

**FIGURE 2:**
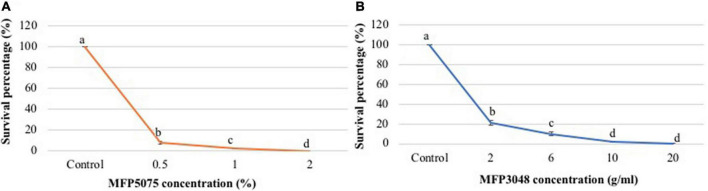
**(A)** Effect of MFP5075 on juveniles of *Globodera rostochiensis*; **(B)** Effect of MFP3048 on juveniles of *G. rostochiensis*. Values represented by similar letters are not significantly different from each other (*p* ≤ 0.05).

Treatment of *G. rostochiensis* J2 with MFP5075 had a very strong and significant impact (*p* ≤ 0.05). Preliminary experiments were conducted at concentrations ranging from 0 to 10% MFP5075. After 24 h of treatment in the 96-well plates, the J2 at all the dilutions except for untreated control did not survive. Subsequently, concentration range was reduced to 0–2%. In this concentration range, after 24 h of treatment in 96-well plates at 0.5 and 1% a 13-and 43-fold reduction in survival was recorded compared to untreated control. However, at 2%, the number of juveniles (J2) which survived dropped to zero (Figure 2A). Therefore, 2% MFP5075 could be considered as the lethal concentration for *G. rostochiensis* J2.

In the case of MFP3048, concentrations used were in the range of 0–20 g/10 ml (w/v) dissolved in water. After 24 h treatment in 96-well plates, in all the replicates, J2 were viable up to 10 g of product. Subsequently, the treatment duration was increased to 48 h with the same concentration range. After 48 h of treatment, percent survival at 2, 6, 10, and 20 g/10 ml dropped to 21.3, 10.2, 2.2, and 0.3%, respectively (Figure 2B). Therefore, 20 g/10 ml (w/v) MFP3048 could be considered as the lethal dose for *G. rostochiensis* J2.

### Attraction assays using 20% (w/v) pluronic gel

**FIGURE 3:**
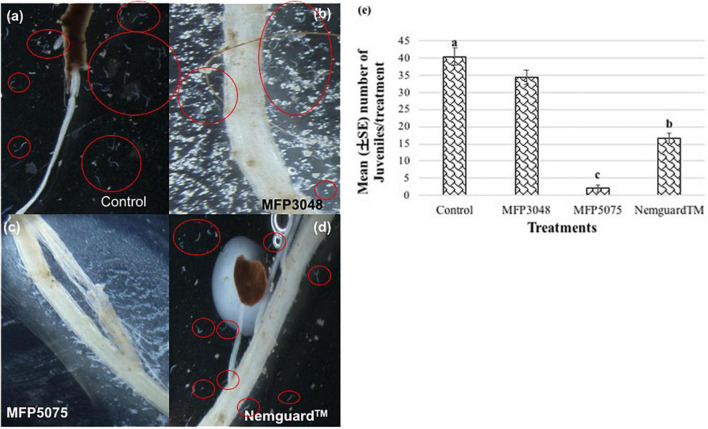
Attraction of *Globodera rostochiensis* J2 toward potato roots observed at 8× magnification: **(a)** Control; **(b)** MFP3048 treatment; **(c)** MFP5075 treatment; **(d)** Nemguard™ treatment; **(e)** Effect of products on the attraction of PCN J2. Values represented in graph by the same letters are not significantly different from each other (*p* ≤ 0.05). Individual and cluster of nematodes are indicated using red circles.

The MFP5075 treatment had a significant effect on PCN J2 in their attraction toward the host roots (Figure 3). There were only 1–2 J2 found attracted to the roots with this treatment (*p* ≤ 0.05). A 20- and 8-fold reduction in number of J2 attracted toward potato roots was observed with MFP5075 (2 ± 0.1) treatment compared to that of untreated control (40.3 ± 2.5) and Nemguard™ (16.7 ± 1.5) treatment, respectively. In the case of treatment with MFP3048, an average of 34.3 ± 2.1 J2 were found attracted to the potato roots, which was not significantly different from the untreated control. All the individuals and clusters of nematodes are indicated using red circles in Figure 3.

### *Globodera rostochiensis* J2 hatching assay from cysts

**FIGURE 4:**
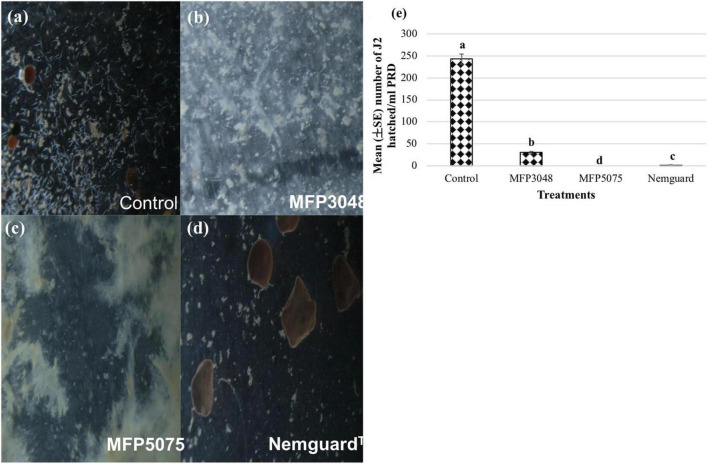
Hatching of *Globodera rostochiensis* J2 observed at 8× magnification in the treatments: **(a)** Untreated control; **(b)** MFP3048 treatment; **(c)** MFP5075 treatment; **(d)** Nemguard™ treatment; **(e)** Effect of products on the hatching of PCN J2 per ml of PRD. Values represented in graph by the same letters are not significantly different from each other (*p* ≤ 0.05).

Approximately 30–35 cysts were treated with MFP5075 (2%), MFP3048 (20 g/10 ml), and Nemguard™ (13.3 mg/L) dissolved in PRD. After 4 weeks of treatment (243.3 ± 11.5), (30 ± 2.6), (0 ± 0), and (1.3 ± 0.6) J2 hatched in untreated control, MFP3048, MFP5075, and Nemguard™ treatments, respectively (Figure 4).

**FIGURE 5:**
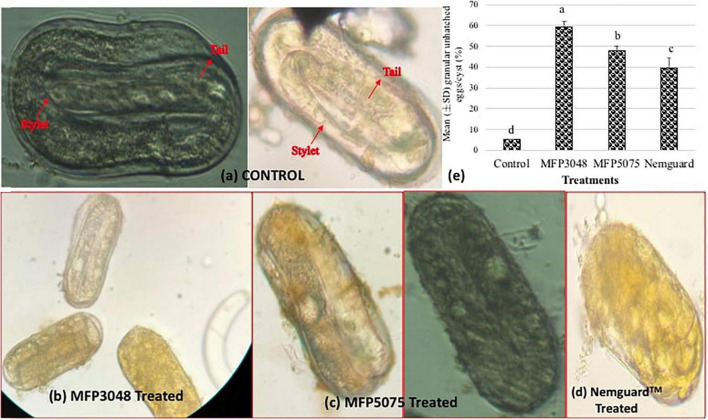
Treatment with products cause granulation in the unhatched eggs. Picture of the unhatched eggs from **(a)** Untreated control; **(b)** MFP3048 treated; **(c)** MFP5075 treated; **(d)** Nemguard™ treated; **(e)** Percentage of granular unhatched eggs/cyst in various treatments. Data shown are mean (±SE) percentage of unhatched eggs that appeared granular, per cyst. Values represented in graph by the same letters are not significantly different from each other (*p* ≤ 0.05).

Approximately 10–15 cysts per treatment were broken open carefully after 4 weeks. Efforts were made to identify the stylet, esophagus and the tail regions of the unhatched juveniles within the cysts. Most of the eggs in the untreated control were found as empty shells. However, many eggs were found granular in the MFP3048 treated cysts when compared to those treated with MFP5075, Nemguard™ treatment, and in the untreated control (Figure 5). Treatment with MFP5075 caused granulation in 47.7% of the unhatched eggs which was higher than that of the eggs that were found granular when treated with Nemguard™ treatment (39.3%). However, the number of granular eggs was highest in the MFP3048 (59.3%) treatment (Figure 5).

Upon transferring the individually treated cysts into normal PRD without products, no hatching in the MFP3048 treated cysts was observed. However, approximately 3–4 and 60–70 J2/ml PRD were found hatched in the MFP5075 and the Nemguard™ treated cysts, respectively.

### Effect of microbial based products on *Globodera rostochiensis* infestation in potato plants

*Globodera rostochiensis* J2 completely killed all the inoculated untreated potato plants in all the trials. The inoculated plants were all found completely dried up, wilted and dead within 1 month of infection. Treatment with the MFP products and Nemguard™ had a very significant impact on the growth of the potato plants (Table 1). Potato plants that were infested with PCN J2 and treated with 1% MFP5075 performed very well regarding all the growth parameters. The MFP5075 (24.7 ± 5.0) treatment enhanced the root growth by 1.6–2.1-fold when compared to Nemguard™ (15.2 ± 2.75) and the MFP3048 (11.5 ± 2.6) treatments. Only root length was measured and recorded in the inoculated control plants, whereas all other growth parameters were not recorded as the plants were all completely dried up and dead. A 2.68-fold increase in root length of MFP5075 treated plants was noted when compared to that of untreated inoculated control plants.

**TABLE 1:** Effect of various treatments on: number of leaves (NL), shoot height (SH), root height (RH), fresh weight (FW), and dry weight (DW) of treated, inoculated, and untreated potato plants.

Treatments	Mean (±SE) Number of leaves (NL)	Mean (±SE) Shoot height (SH) (cm)	Mean (±SE) Root length (RL) (cm)	Mean (±SE) Fresh weight (FW) (g)	Mean (±SE) Dry weight (DW) (g)	Mean (±SE) Number of cysts/plant
Inoculated control	0 ± 0 c	0 ± 0 c	9.2 ± 0.7 c	0 ± 0 c	0 ± 0 c	35 ± 5 a
10 g MFP3048	19.7 ± 1.2 a	41.3 ± 4.2 a	11.5 ± 2.6 b	5.7 ± 1.6 b	0.8 ± 0.1 b	3.3 ± 0.6 c
1% MFP5075	22 ± 6.6 a	41.7 ± 5.5 a	24.7 ± 5.0 a	10.0 ± 2.9 a	2.1 ± 0.1 a	5.3 ± 1.5 c
Nemguard™	21.7 ± 3.5 a	34.7 ± 9.8 b	15.2 ± 2.75 b	6.6 ± 0.7 b	0.9 ± 0 b	23.7 ± 1.5 b

Values represented in the table by the same letters are not significantly different from each other (p ≤ 0.05).

MFP5075 treatment also had a significant effect on FW, DW, and SH. The SH in the plants treated with MFPs was 1.2-fold greater compared to that of the Nemguard™ treatment. However, MFP5075 had the largest impact on plants in terms of both FW (10.0 ± 2.9 g) and DW (2.1 ± 0.1 g) compared to other treatments. The increase in FW was 1.8- and 1.5-fold for MFP 5075 compared to MFP3048 (5.7 ± 1.6 g) and Nemguard™ (6.6 ± 0.7 g) treatments. The increase in DW was 2.6- and 2.3-fold compared to the MFP3048 (0.8 ± 0.1 g) and Nemguard™ (0.9 ± 0 g) treatments.

**FIGURE 6:**
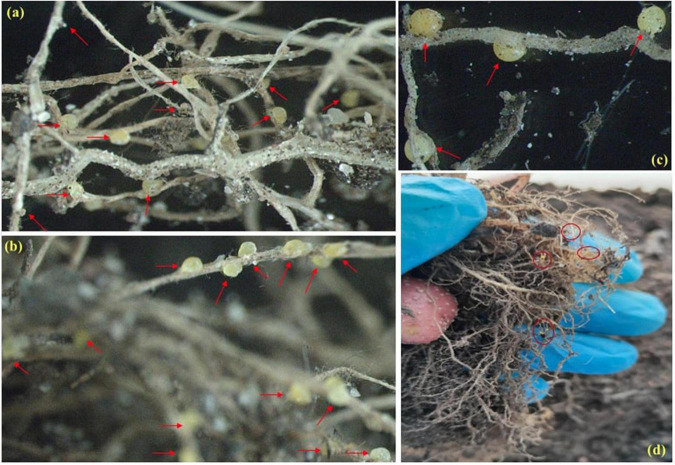
Images of cysts developed on potato roots (2–10× magnification) in: **(a)** Untreated control; **(b)** Nemguard™ treated; **(c)** MFP5075 treated; and **(d)** MFP3048 treated. Red arrows in the pictures indicate the cysts or females developed on each root system.

The most interesting observation was made in relation to the number of females/cysts developed per root system (Table 1 and Figure 6); the cysts developed on each root system are indicated using red arrows in Figure 6. The plants treated with both MFPs had less PCN infestation with least number of cysts noted in the MFP3048 (3.3 ± 0.2) treatment when compared to MFP5075 (5.3 ± 1.5), Nemguard™ (23.7 ± 1.5), and the untreated control (35 ± 5) (Table 1). The infection was reduced by 10.6- and 6.6-fold with MFP3048 and MFP5075 treatments compared to untreated control plants, respectively. This reduction was 7.2- and 4.5-fold when compared to the treatment with the commercial nematicide Nemguard™ treatment.

### Garden soil treatment with microbial fermentation products, deoxyribonucleic acid extraction, and sequencing

Garden soil patches were treated with MFP5075, MFP3048, Nemguard™, Vydate™, and there was an untreated control. A dominant phylum detected along with Nematoda was Apicomplexa. A few other minor organisms belonging to the phyla Arthropoda, Annelida, Basidiomycota, Ciliophora, Cercozoa, Ascomycota, Rotifera, and Mucoromycota were also detected, however, they were not in the interest of our study.

The ternary plot is shown in Figure 1b. In the plot the three vertices are represented by the three samples or groups. Circles represent dominant taxa and the size of circles is proportional to the relative abundance of the phyla. The sample or group to which the circle is closer, is represented in higher abundance in the respective phylum. The plot clearly displayed that the phylum Nematoda was dominant in soil samples treated with MFP5075 followed by the soil samples treated with MFP3048. Members of the phylum Nematoda were least abundant in samples treated with the commercial nematicide Nemguard™.

Heatmaps representing the abundances of the top nine nematode orders in treatment groups are shown in Figure 1c. The identified nine dominant nematode order were Dorylaimida, Triplonchida, Tylenchida, Enoplida, Monhysterida, Chromadorida, Dorylaimia, Araeolaimida, and Rhabditida. Among them Dorylaimida, Triplonchida, and Dorylaimia mostly contain free-living terrestrial and freshwater species. The orders Monhysterida, Enoplida and Araeolaimida contain marine free living nematodes; Tylenchida are mostly parasites of insects or plants and Rhabditida is an order of free-living, zooparasitic, and phytoparasitic microbivorous nematodes living in soil. From the heat map it is evident that, the nematodes belonging to the order Dorylaimida and Triplonchida were abundant in the untreated control, Vydate™ and MFP5075 treated samples whereas, they were least abundant in case of MFP3048 and Nemguard™ treated soils. A significantly different observation was made regarding the order Tylenchida, which were highly abundant in all the treatments except in the soils treated with the chemical nematicide Vydate™. Similarly, the Vydate™ treatment completely reduced the abundance of the nematodes belonging to the orders Monhysterida, Chromadorida, Dorylaimia, Araeolaimida, and Rhabditida. The heat map gives an overall indication, that out of the nine nematode orders identified, seven were abundant in the soils treated with MFP5075 and six of them were found abundant in the soils treated with MFP3048 and Nemguard™. The lowest nematode abundance was recorded in the soils treated with the chemical nematicide Vydate™, which had only three out of the nine orders identified.

## Discussion

Although there are many reports on applications of various products to control PCN infestation, most of them still remain incomplete due to certain limitations. Some of the nematicides that are currently in use are synthetic based and therefore highly toxic for the environment (Feist et al., 2020).

The bioassays clearly indicated the nematicidal potential of the products by causing 100% mortality at very low concentrations. Similar observations were noted when the root-knot nematode, *M. javanica* was treated with MFP5075 (Pulavarty et al., 2020, 2021a). Application of pluronic gels facilitates easy observation of juvenile movement toward host roots (Wang et al., 2009; Sasaki-Crawley et al., 2012; Pulavarty et al., 2021a). The *in vitro* bioassays that are reported here on PCN juvenile mortality, J2 hatching from the cysts and attraction towards host roots, give a clear indication that the product MFP5075 has an impact on the activity of the PCN. It has the potential to cause mortality directly to J2 as 2% of its concentration caused 100% mortality within 24 h. Similarly, the attraction assays also revealed the potential of the product as it completely blocked the movement of J2 toward the host roots by killing the J2. Similar effect was noticed while treating *M. javanica* J2 with the same product (Pulavarty et al., 2021a). This observation indicates that the application of MFP5075 to the PCN infected soil, it could potentially supress the infection by causing death of the juveniles that are available in the soil and also cease the hatching process. Potentially, if a product can cease the hatching process then it has the ability to interfere with the life cycle of the nematode and could stop the release of the next generation of the PCN (Zakaria et al., 2013). Therefore, in the course of time the nematodes could be completely eradicated from the soils. However, in such cases, the shelf-life of the applied products must be thoroughly investigated so as to ensure its prolonged effect on nematodes.

The observation on egg viability or juvenile integrity within the eggshells, suggests the *in ovo* necrotic potential of the MFPs, especially, MFP3048, as it caused the highest number of granular unhatched eggs. This gives an indication that onset of favorable conditions might not promote the hatching process as the product possibly is causing morphological necrosis within the egg itself. This again indicates that this product interferes with the PCN life cycle by irreversibly inhibiting the hatching process. This loss of J2 integrity clearly depicts the necrosis and suggests a potential mode of action of MFP3048. This could possibly explain the 8.1-fold reduction in J2 hatching, when cysts were treated with MFP3048 when compared to that of untreated control. The *in ovo* exposure to MFP3048 caused an impairment in J2 hatching even after transferring the cysts to normal root diffusates. MFP5075 treatment also caused granulation in the *G. rostochiensis* eggs but this was lower than that caused by the MFP3048 treatment, with only a few J2 observed to hatch when the cysts were put back into normal PRD. However, the Nemguard™ treatment caused least granulation in the unhatched eggs, therefore the cysts when treated with this nematicide resulted in the hatching of juveniles following the onset of normal conditions. Similar observations were reported in *G. pallida*, when its eggs were treated with aldicarb, as there was no evidence of irreversible inhibition of the hatching process (Feist et al., 2020). Conversely, treatment with Fluopyram and Abamectin was reported to cause heavy granulation within the eggs of *G. pallida* and therefore reduced the J2 hatching process due to morphological disruption of eggs (Feist et al., 2020). All these nematicides are synthetic agrochemicals and have been reported to be highly toxic to birds, mammals, and aquatic organisms (European Food Safety Authority, 2013).

Prophylactic treatment with the MFPs clearly showed potential of plant growth promotion. The potato plants treated with MFP5075 had the highest biomass accumulation compared to the plants in other treatments. These observations were in agreement with previous findings that were reported on tomato plants (Pulavarty et al., 2021a). MFP3048 also contributed toward growth of potato plants compared to those in the untreated control but had less biomass accumulation compared to that in the MFP5075 treatment. However, nematode suppression potential was more evident in the MFP3048 treatment (Table 1), as the roots treated with this product had the least number of females or cysts developed on them compared to any other treatment. The reason for this could be directly related to the granulation that was caused in eggs treated with this product. Treatment of plants with this product might have increased the number of granular eggs thus reducing J2 hatching therefore interrupting the lifecycle of PCN and ultimately reducing the cyst formation on the roots. The untreated potato plants were all dead with highest number of cysts or females developed in their roots, whereas, the MFP treated plants not only had higher biomass accumulation but also were found to have the least number of cysts or females developed on their roots compared to the plants in the untreated control and the commercial nematicide treatment (Nemguard™). This could be due to the presence of essential micronutrient, bacterial fermentation extracts and Cu component within these products that are contributing toward plant growth promotion (Pulavarty et al., 2020). Aires et al. (2009) reported *G. rostochiensis* suppression following treatment with extracts of Brassicacea plants. The study showed that maximum nematode suppression was noted when the plants that were treated with extracts of watercress, cauliflower and *Brassica rapa* (Aires et al., 2009). However, the effect of these extracts on other soil nematodes, EPN and on potato plant growth have not been reported.

Prior to large scale field application, a pilot scale trial needs to be performed to study the nematode abundance in the soils after application of any products (Abawi and Widmer, 2000; Briar et al., 2007; Oka, 2010). This kind of analysis would provide an indication on what could possibly happen to non-target beneficial nematodes in the soil. This would certainly help to prevent causing any long term damage to natural soil ecology (Abawi and Widmer, 2000; Pulavarty et al., 2021b). Therefore, in this study with Alltech MFPs we tried to fill the knowledge gaps by studying their impact on EPN (Pulavarty et al., 2020), other soil nematodes and on plant growth promotion were assessed in a small pilot scale study.

Our results in relation to nematode abundance in soil indicated that the application of the MFPs had a very similar effect to that in the untreated control and the Nemguard™ treatment. These observations when combined with that of the previous results reported in terms of the MFPs effect on the beneficial EPN (Pulavarty et al., 2020), indicate that the MFP formulations could be safe for the environment. However, these results are very preliminary and further repetition and large-scale field trials are essential to validate the reported results on nematode biodiversity.

Compiling all the findings, it could be concluded that under the conditions of this study the MFPs, which are organic based soil health products, displayed nematicidal properties against *G. rostochiensis*. These products reduced PPN infestations in the host plants and displayed a positive impact on the beneficial EPN (Pulavarty et al., 2020, 2021a). Application of these products could potentially improve the potato yield and help toward enhancing the global potato production. Therefore, subsequent large scale field applications should be performed to further confirm the findings of this work and potentially contribute toward the sustainable management of PCN.

## Data availability statement

The data presented in this study are deposited in the NCBI repository, accession numbers SAMN30418779, SAMN30418780, SAMN30418781, SAMN30418782, SAMN30418783, SAMN30418784, SAMN30418785, SAMN30418786, SAMN30418787, and SAMN30418788.

## Author contributions

AP: planning, execution, statistical analysis, and manuscript drafting. AS: performing experiments and helping AP. DS: performing nematode biodiversity studies. JM: bioinformatic analysis of nematode biodiversity studies. KH: manuscript editing, mentoring, and funding the research. TK-D: manuscript editing, mentoring, and through supervision. All authors contributed to the article and approved the submitted version.

## Footnotes

1
https://atlasbig.com/en-ie/countries-by-potato-production

2
http://www.arbsilva.de/

3
http://www.drive5.com/usearch/manual/uchime_algo.html

4
https://drive5.com/usearch/manual/chimeras.html

5
http://drive5.com/uparse/

6
http://qiime.org/scripts/assign_taxonomy.html

7
http://www.arb-silva.de/

8
http://www.drive5.com/muscle/
